# Evaluation of phosphorus fertilizer sources and nitrogen optimization for wheat and tef in Ethiopia’s central highlands

**DOI:** 10.1038/s41598-025-34369-6

**Published:** 2026-01-03

**Authors:** Yalemegena Gete, Beza Shewangizaw, Kenzemed Kassie, Shawl Assefa, Tadele Amare, Tesfaye Feyisa, Getaneh Shegaw, Lisanu Getaneh, Dejene Mamo, Getachew Lema, Genet Taye

**Affiliations:** 1 Amhara Agricultural Research Institute, Debre Birhan Agricultural Research Center, P.O. Box 112, Debre Birhan, Ethiopia; 2 Amhara Agricultural Research Institute, Adet Agricultural Research Center, P.O. Box 08, Bahir Dar, Ethiopia; 3 Amhara Agricultural Research Institute, P.O. Box 527, Bahir Dar, Ethiopia

**Keywords:** DAP, NPS, TSP, North Shewa, Ecology, Ecology, Environmental sciences, Plant sciences

## Abstract

The application of appropriate fertilizer sources and the optimization of nitrogen management are key strategies for increasing crop yield and nutrient use efficiency. An on-farm experiment was conducted in five districts of the North Shewa Zone, Amhara Region, Ethiopia, to evaluate three phosphorus sources (NPS, DAP, and TSP) and nitrogen application times (100% and 75% of the recommended rate, with split applications) for wheat and tef production. The experiments for bread wheat were conducted on contrasting soil types (Cambisols, heavy Vertisols, and light Vertisols), whereas the experiments for tef were conducted on heavy Vertisols. A randomized complete block design was used, with a farm considered a replication (only a single replication with all treatments was planted at a farm). Data on growth and yield were analyzed using R software version 4.3. All phosphorus sources significantly increased yields compared to the control, with wheat yields increasing from 1,898 to 4,640-5,360 kg ha^-1^ and tef from 1,376 to 2,382-2,591 kg ha^-1^. Notably, the 75% N rate with split application improved the agronomic efficiency of nitrogen (AEN) by 38.8% and the nitrogen use efficiency (NUE) by 19.5% compared with the previously recommended two-split applications, suggesting a cost-effective and efficient N management approach. Farmer preferences, assessed via Likert scales, aligned with the observed biological yield trends. These findings suggest that NPS, DAP, and TSP perform similarly from an agronomic perspective, and fertilizer choice can be guided by local availability and cost. Reduced, split nitrogen applications offer a cost-effective way to improve wheat and tef productivity and nutrient use efficiency, supporting sustainable fertilizer management.

## Introduction

Agricultural productivity in developing countries such as Ethiopia is closely linked to the effective use of fertilizers^1^. In a country where agriculture is the backbone of the economy and rural livelihoods, adopting sustainable and efficient agricultural practices is essential to meet food security challenges^2,3^. Bread wheat and tef (Eragrostis tef), as staple crops in central Ethiopia, are crucial for national food security and serve as major sources of income^4,5^, highlighting the importance of improved nutrient management to increase productivity and economic resilience. Despite the widespread use of nitrogen and phosphorus fertilizers over the past five decades^6^, low productivity persists due to nutrient deficiencies, poor soil fertility, and suboptimal fertilizer use^7^. Among these nutrients, nitrogen remains particularly critical, as it is often the most yield-limiting nutrient^8,9^. Therefore, optimizing nitrogen use not only in terms of application rates but also in terms of timing and methods is essential for increasing nitrogen use efficiency (NUE), reducing input costs, and maximizing crop yields^10,11^.

Triple Superphosphate (TSP) was the main phosphorus fertilizer used globally until the mid-1970s^12^ and has been used in Ethiopian research fields for the past five decades. Since then, diammonium phosphate (DAP) has been the dominant phosphorus fertilizer in Ethiopia for decades because of its combined supply of nitrogen and phosphorus^13^. Since 2015, Ethiopia has introduced NPS fertilizer, which is based on soil fertility mapping conducted by the EthioSIS program, to address sulfur deficiencies^14^. However, subsequent research confirmed that only nitrogen and phosphorus are yield-limiting nutrients^9,15^. In addition to these changes in fertilizer sources, increasing population pressure and declining soil fertility steadily increased the demand for P-based fertilizers. Therefore, over the past decade, there has been increasing pressure to identify the most suitable phosphorus source for Ethiopian agriculture.

Compound fertilizers like NPS and DAP provide multiple nutrients simultaneously and can be effective when soil nutrient deficiencies match their composition^16^. However, their fixed nutrient ratios may not always align with site-specific soil needs^17,18^. In contrast, single-nutrient fertilizers such as TSP deliver one primary nutrient and enable customized nutrient management^19^. Phosphorus, the key nutrient supplied by these fertilizers, is critical for plant metabolism, particularly in energy transfer and nucleic acid synthesis^20^. Historically, DAP was the main phosphorus fertilizer in Ethiopia until soil mapping efforts by EthioSIS encouraged the adoption of blended fertilizers such as NPS, NPSB, and NPSBZn^14^ to address broader nutrient deficiencies and improve crop yields^21^. Nitrogen, a primary essential nutrient for crops, is often lost through leaching, volatilization, and denitrification, reducing its availability and limiting yields^22^. In Vertisols, where nitrogen is the primary yield-limiting nutrient, nitrogen management is essential for maximizing yield and sustaining productivity^23,24^.

Furthermore, to strengthen the interpretation of the field-based biological yield data, farmer perception information was gathered through a participatory evaluation, providing practical insights that helped validate treatment performance under real farming conditions. The objective of this research was to evaluate the agronomic and economic performance of different phosphorus fertilizers (TSP, NPS, DAP) through on-farm trials in central Ethiopia, with a particular focus on nitrogen optimization. The findings can inform cost-effective, nutrient-efficient management for improving wheat and tef productivity, contributing to food security and sustainable agriculture.

## Materials and methods

### Description of the study area

**Fig. 1:**
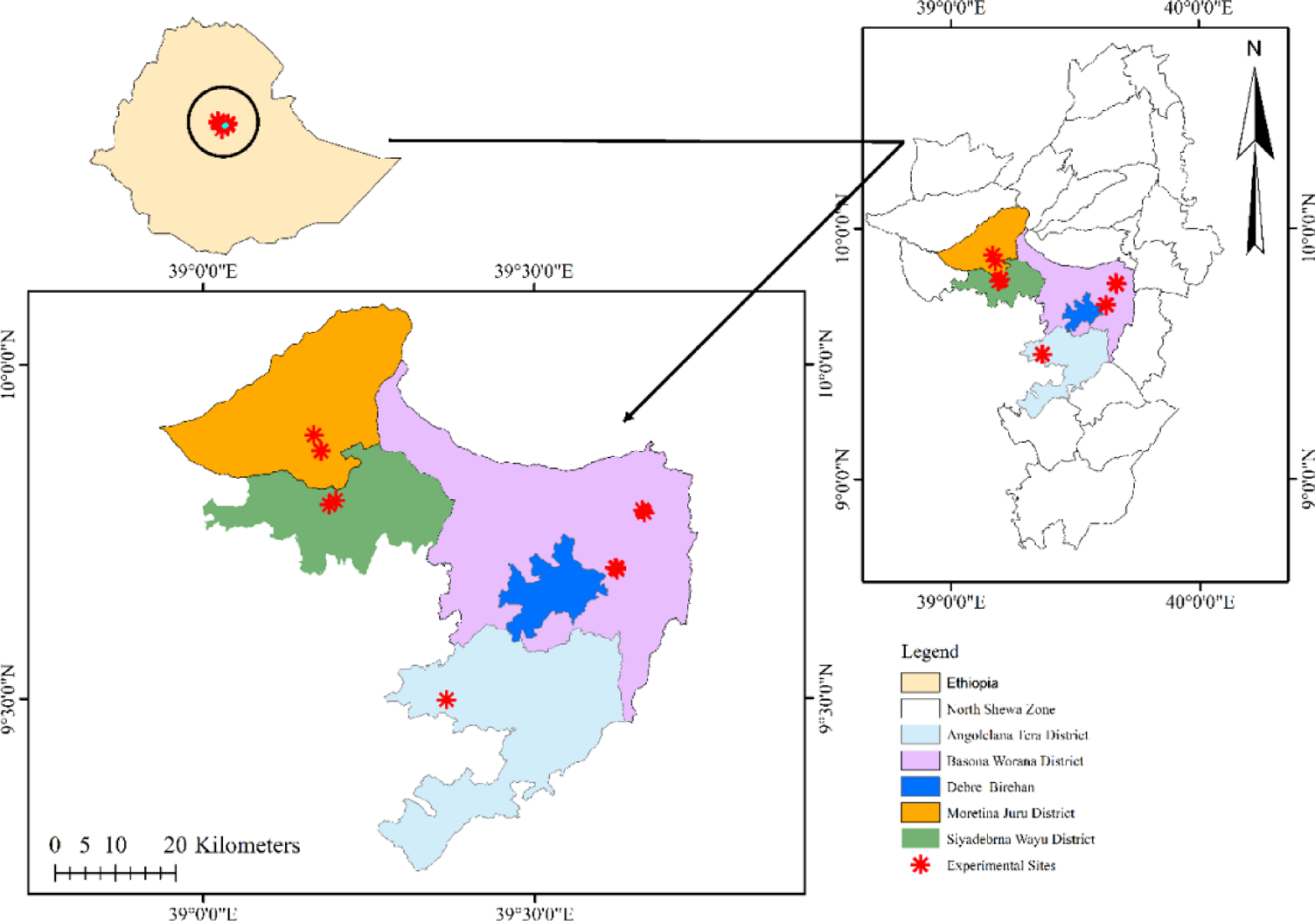
Map of the study area. Source: The map was generated in ArcGIS 10.5 using shapefiles downloaded from this link: [https://open.africa/dataset/ethiopia-shapefiles]^25^.

The on-farm experiment was carried out in five districts of the North Shewa Zone, Amhara Region, Ethiopia: Debre Birhan, Basona Werana, Angolelana Tera, Siyadebrina Wayu, and Moretina Jiru, encompassing 14 farmers’ fields, of which 10 were wheat fields, and 4 were tef fields (Fig. 1). Wheat experiments were conducted under a range of agroecological conditions (highland and mid-highland), farming practices, and soil types, i.e., light and heavy Vertisols and Cambisols. While tef trials are being conducted in heavy Vertisols. The study was conducted in Ethiopia’s central highlands across major wheat and tef production areas located at altitudes ranging from 2626 to 2995 m above sea level. The region is characterized by a unimodal rainfall pattern, with rains typically occurring from June to September and peak precipitation occurring in July and August during the cropping season. All districts practice a mixed farming system that integrates both crop and livestock production.

### Experimental design and treatments

Each farmer’s field served as a replicate in a randomized complete block design, with all treatments randomly assigned within each field. Ten farms were used for wheat trials and four for tef trials. In this setup, each farmer’s field received all the treatments, enabling the evaluation of treatment effects while considering interfarmer differences. The research evaluated phosphorus fertilizer sources, including NPS, DAP, and TSP, combined with nitrogen optimization options. The recommended rate of P₂O₅ was applied at planting for each crop, using equal amounts from each phosphorus source. The detailed treatment structure is provided in Table 1. The treatments were randomized within each farmer’s field and allocated to individual plots. Each plot measured 5 m in width and 4 m in length, with a 1 m space between adjacent plots.

**Table 1:** Description of the treatments.

#	Treatment name	Treatment application
T1	Negative control	Without fertilizer
T2	NPS + 100% RN	100% recommended rate of P_2_O_5_ from NPS at sowing. 100% recommended rate of N (1/2 at sowing + 1/2 at tillering).
T3	DAP + 100% RN	100% recommended rate of P_2_O_5_ from DAP at sowing. 100% recommended rate of N (1/2 at sowing + 1/2 at tillering).
T4	TSP + 100% RN	100% recommended rate of P_2_O_5_ from TSP at sowing 100% recommended rate of N (1/2 at sowing + 1/2 at tillering).
T5	TSP + 100% RN	100% recommended rate of P_2_O_5_ from TSP at sowing. 100% recommended rate of N (0 at sowing + 2/3 at tillering + 1/3 at jointing).
T6	TSP + 75% RN	100% recommended rate of P_2_O_5_ from TSP at sowing. - 75% recommended rate of N (0 at sowing + 1/2 at tillering + 1/2 at jointing).

### Fertilizer application rates

Fertilizer applications for wheat were determined at the district level and aligned with the official NP recommendations:138/69 kg N/P₂O₅ ha^− 1^ for low-input sites^26^ and 253/69 kg N/ P₂O₅ ha^− 1^ for high-input sites^27^. Since no fertilizer recommendations are available for medium-input areas, an intermediate rate of 192/69 kg N/P₂O₅ ha^− 1^ was applied for Cambisols. This rate represents a logical interpolation between the established low- and high-input recommendations and is further supported by previous NPKS refinement work in the district (unpublished progress report), which indicated optimal yield ranging from 176 to 222 kg N ha^− 1^. The intermediate rate also facilitates secondary analysis of the consistency of P source performance across contrasting N levels. For tef, which is grown in the Siyadebrina Wayu and Moretina Jiru districts, nutrient application rates were standardized at 69 kg P₂O₅ ha^− 1^ and 170 kg N ha^− 1^, respectively^28^. The detailed nutrient application rates are summarized in Table 2.

**Table 2:** Nutrient application rates for each district and crop type.

District	Test crop		Description	Nutrient application rate (kg ha^− 1^)			
				Wheat		Tef	
	Wheat	Tef		*N*	*P*_2_O_5_	*N*	*P*_2_O_5_
Basona Werana	✓	–	Medium-input area	192	69	–	–
Debre Birhan	✓	–	Medium-input area	192	69	–	–
Angolelana Tera	✓	–	Low-input area	138	69	–	–
Moretina Jiru	✓	✓	High-input area	253	69	170	69
Siyadebrina Wayu	✓	✓	High-input area	253	69	170	69

### Crop management practices

The varieties used were Dendea for bread wheat and Dega for tef. The planting dates and other practices for both wheat and tef were followed by the recommendations for the specific area. In the highland agroecological zone, wheat was planted between July 8 and 11, 2024, whereas in the mid-agroecological zones, it was planted from July 23 - 24, 2024. To address site-specific conditions, different planting methods were used on the basis of the soil type. In Vertisols, a broad bed and furrow (BBF) system was adopted to enhance drainage and mitigate waterlogging issues. Row planting was implemented on Cambisols to suit their drainage and agronomic requirements. While tef was sown by the broadcast method. Weeding practices differ significantly across farmers’ fields and are influenced by weed type and intensity. A single application of herbicide called Pallas was used for all the experimental units. This was supplemented with one round of hand weeding. In fields with high weed pressure or specific weed challenges, a second round of hand weeding was conducted to ensure optimal weed control.

### Agronomic data collection

The plant height at maturity was measured in centimeters from the soil to the tip of the spike (excluding awns) as the average of ten plants per plot. The number of fertile tillers was estimated by counting the number of plants with heads or spikes in a sample of ten plants per plot at physiological maturity. The average lengths of ten randomly selected spikes and panicles of the main tiller were measured during physiological maturity in cm from base to tip, excluding the awn for wheat and tef, respectively. Biomass yield was calculated by weighing the total air-dried aboveground biomass harvested from the net plot areas and rows and then expressed in kilograms per hectare. The grain yield weight of the grains was calculated from harvested wheat plants in the net plot areas after sun drying, threshing, and cleaning; the weight was then converted to kilograms per hectare, and the grain yield was adjusted to a 12.5% moisture basis. Farmer and expert perception data were collected at the full heading stage to complement the grain and biological yield results. A total of 36 farmers and 5 experts provided perception data on the phosphorus source treatments using a structured Likert-scale assessment. Moreover, nitrogen use efficiency parameters such as the agronomic efficiency, recovery efficiency, and utilization efficiency of wheat were calculated following the methods of^29^.

I. Agronomic efficiency is the grain yield gained per unit of nutrient applied and is calculated as follows: AE (kg kg⁻¹) **=** (Gf - Gu)/Na, where *Gf* = yield with fertilizer, *Gu* = yield without fertilizer, and *Na* = nutrient applied.
II. Apparent recovery efficiency is the nutrient uptake gained per unit of nutrient applied, calculated as ARE (%) = [(Nf - Nu)/Na] × 100, where *Nf* = uptake with fertilizer, *Nu* = uptake without fertilizer, and *Na* = nutrients applied.
III. Nutrient use efficiency is the grain yield increase per unit of nutrient taken up, expressed as the product of recovery efficiency and physiological efficiency: NUE (kg kg⁻¹) = RE × PE.

### Soil sampling preparation and analysis

Pre-sowing, soil samples were collected from 10 locations within the experimental field (0 - 20 cm depth) via a zigzag pattern to form a composite sample. After mixing, a 1 kg subsample was taken, air-dried, and ground to pass through sieves (0.5 mm for nitrogen, 2 mm for other tests)^30^. The soil pH was measured with a pH meter in a 1:2.5 soil-to-water suspension^31^. Organic carbon was measured via the Walkley-Black method, and total nitrogen was determined via the Kjeldahl method^32^. Available P was extracted with a sodium bicarbonate solution at pH 8.5, following the procedure described by^33^. The soil texture was determined via the Bouyoucos hydrometer method^34^. The cation exchange capacity (CEC) was determined via the 1 M ammonium acetate method at pH 7^35^.

### Data analysis

The effects of the various treatments on the wheat and tef yield were tested. The collected data were subjected to analysis of variance (ANOVA) via the R software program version 4.3^36^. Upon the existence of a significant difference for ANOVA (*p* < 0.05), further mean separation analysis was performed using the honest significance difference (HSD) at a 5% level of significance^37^. Graphical analysis was also used to evaluate the response. The Likert scale data were analyzed graphically and summarized using descriptive statistics (mean ± standard deviation). Wheat yield data were analyzed using a linear mixed-effects model:Y_ijk_=µ + Trt_i_ + Soil_j_ + (Trt*Soil)_ij_ + Rep_k_ + Ɛ_ijk_ Where Y_ijk_ represents the wheat yield (kg ha⁻¹) for the ith treatment, jth soil type, in the kth replication (farmer), µ is mean wheat yield (kg ha^-1^), Trt_i_ is the fixed effect of the ith treatment, Soil_j_ is the fixed effect of the jth soil type, Rep_k_ is the random effect of the kth replication (farmer), ε_ijk_ is the residual error term. The tef yield data were analyzed using the following linear mixed model: Y_ij_= µ + Trt_i_ + Rep_j_ + Ɛ_ij_ Where Y_ij_ is the tef yield (kg ha⁻¹) for the ith treatment at the jth replication (farmer field, µ is the mean tef yield (kg ha^-1^), Trt_i_ is the fixed effect of the ith treatment, Rep_j_ is the random effect of the jth replication (farmer field), and ε_ij_ is the residual error term.

## Results and discussion

### Physicochemical properties of soils at the experimental sites

The pre-sowing physical and chemical soil properties are summarized in Table 3. The pH values at the study sites were similar for both the wheat and the tef farms, with mean values of 6.13 and 6.12, respectively. According to^38^ , this range is classified as moderately acidic to neutral, which is generally optimal for most crops, as it supports nutrient availability and microbial activity, creating favorable conditions for crop establishment^39^. The mean cation exchange capacity (CEC) was 35.97 cmolc kg⁻¹ for wheat farms and 39.13 cmolc kg⁻¹ for tef farms, indicating high nutrient retention potential, according to^31^. This suggests a strong ability of the soils to retain essential nutrients throughout the growing season^40^. The soil organic carbon (SOC) content was greater on wheat farms, 1.11%, compared to 0.82% SOC in tef farms. Similarly, the mean total nitrogen (TN) content was slightly greater in the wheat soils (0.12%) than in the tef soils (0.09%). According to^31^, both SOC and TN levels fall within the very low to low range, which could result in a positive response to applied nitrogen fertilizer. These low levels of SOC indicate limited biological activity and suboptimal soil structure, which can hinder moisture retention^41^. Similarly, low nitrogen levels indicate a deficiency that limits wheat yield, leading to stunted growth, poor tillering, and reduced grain production^27^. Available phosphorus was also higher on wheat farms (15.30 ppm) compared to tef farms (11.85 ppm), placing it in the medium category according to^42^. However, the variability in phosphorus availability across the sites may lead to inconsistent root development and uneven early growth, contributing to the observed differences in wheat yield. The soil texture of the experimental sites was classified as clay; however, the proportions of sand, clay, and silt varied, which may be the reason for the observed numerical differences in wheat yield with soil type. Overall, the wheat experimental sites exhibited slightly better soil fertility characteristics compared to tef sites at planting, although all the experimental sites were within the typical range for rainfed highland areas in Ethiopia. Overall, no statistical comparison was conducted between wheat and tef farm soils due to different sample sizes and crop-specific management objectives.

**Table 3:** Mean soil physicochemical properties of the experimental site before planting (mean ± standard deviation).

Test crop	pH (1:2.5)	CEC (cmol_c_ kg^− 1^)	SOC (%)	OM (%)	T.*N*(%)	Av.*P*(ppm)	Texture			Textural class
							Sand (%)	Clay (%)	Slit (%)	
Wheat farms (*n* = 10)	6.13 ± 0.47	35.97 ± 5.12	1.11 ± 0.41	1.91 ± 0.70	0.12 ± 0.04	15.3 ± 9.18	17.4 ± 6.04	60.8 ± 14.43	21.8 ± 9.02	Clay
Tef farms (*n* = 4)	6.12 ± 0.22	39.13 ± 4.27	0.82 ± 0.07	1.42 ± 0.12	0.09 ± 0.008	11.85 ± 5.28	12 ± 1.63	74.5 ± 2.52	13.5 ± 1.91	Clay
State of the soil	Slightly acidic	High	Very low	Low	Very low to low	Medium				
References	^38^	^31^	^31^	^43^	^31^	^42^				

### Farmers’ perception of the response of wheat and tef to different phosphorus fertilizer sources at the vegetative stage

**Fig. 2:**
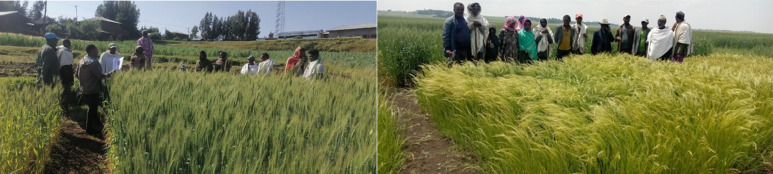
Field performance of wheat and tef crops during the green stage evaluation.

**Fig. 3:**
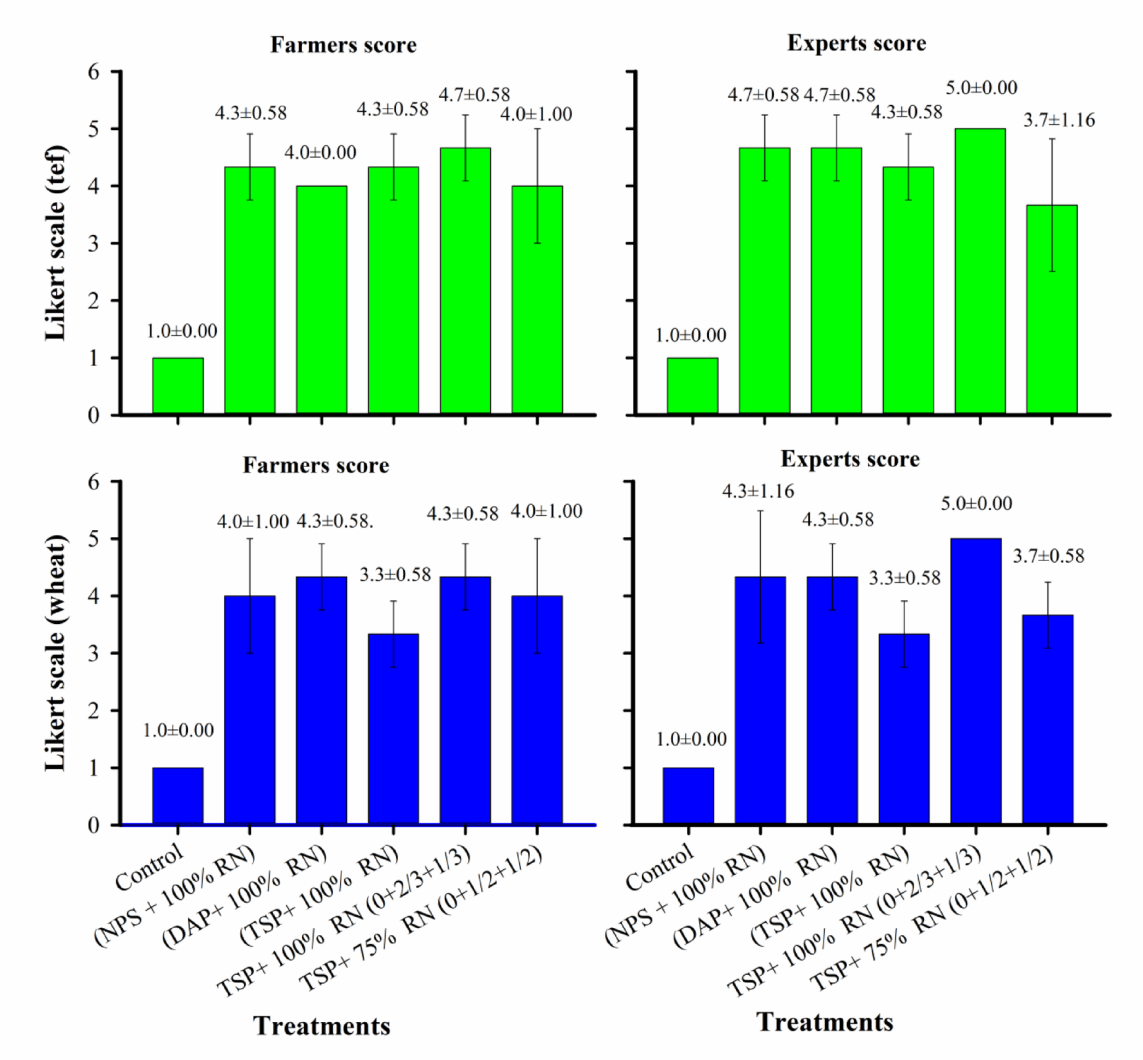
Likert scale scores of farmers and experts on wheat and tef crops during the green stage (mean ± standard deviation).

To support the biological yield data, we collected perception-based evaluations from 36 farmers and a team of researchers across Moretina Jiru, Siyadebrna Wayu, and Basona Werana districts during the green stage of wheat and tef. Participants scored the treatments using a 1 - 5 Likert scale, where “very good” (5), “good” (4), “acceptable” (3), “poor” (2), and “very poor” (1), based on visual criteria such as spike length, greenness, tiller number, and plant height (Figs. 2 and 3). All fertilized treatments received scores above 3, while the unfertilized control consistently scored 1, reflecting clear positive perceptions of fertilizer application. The highest-ranked treatment, 100% recommended P₂O₅ from TSP combined with 100% recommended N applied in split doses (0 at sowing + 2/3 at tillering + 1/3 at jointing), corresponded with the highest biological yields, indicating strong agreement between visual assessments and measured crop performance. The second-ranked treatment, 100% P₂O₅ from DAP + 100% recommended N, also showed alignment between farmer/expert scores and yield data. Treatments with NPS were positively perceived but ranked slightly lower, consistent with their moderate yield responses. Even the 75% recommended N treatment received improved scores over the control, matching its intermediate yield performance. Overall, the perception rankings closely reflected the measured biological outcomes, highlighting the reliability of farmer and expert assessments in reflecting treatment effectiveness.

### Wheat and tef growth parameters

The statistical analysis of variance revealed that all the growth and yield parameters of wheat and tef were highly significantly affected by the treatments (*p* < 0.001) (Table 4). Fertilizer application significantly increased the wheat and tef growth parameters, but there were no significant differences among the fertilized treatments (Table 5). These findings indicate that different phosphorus fertilizer sources (NPS, DAP, and TSP), as well as nitrogen optimization treatments, equally affected crop growth parameters. Numerically, the highest plant heights of wheat (99.7 cm) and tef (97.3 cm) were recorded from the recommended rate of P_2_O_5_ from the TSP + 100% recommended N treatment (0 at sowing + 2/3 at tillering + 1/3 at jointing), whereas the lowest values were recorded from the control treatment. This result reflects that N and P are limiting nutrients, that fertilizer application is mandatory instead of a source, and that nitrogen optimization is very important for promoting wheat and tef growth. Similarly^44^, reported no significant difference between different P fertilizer sources in terms of the number of seeds per spike and the number of fertile tillers, but they reported an increase in maize crops with P fertilization. Additionally^45^, confirmed that P source fertilizers did not affect maize plant height. The results of this experiment clearly illustrate that by using efficient split application of nitrogen, we can save 25% of the recommended N (cost of N is reduced) without affecting wheat and tef growth parameters. Similarly^46^, reported that the application of nitrogen in two splits (1/3 at planting and 2/3 at tillering) resulted in greater plant heights and fertile tiller numbers of durum wheat. Similarly^47^, reported increases in the plant height and tiller number of bread wheat due to three split applications of nitrogen.

**Table 4:** Summary of analysis of variance and probabilities of the effects of treatments on the growth and yield parameters of wheat and tef (F value).

Wheat								Tef							
Source of variation	Df	Plant height	Spike length	Fertile tiller	Grain yield	Biomass yield	HI	Source of variation	Df	Plant height	Panicle length	Fertile tiller	Grain yield	Biomass yield	HI
Trt	5	22.64***	18.99***	17.54***	38.05***	47.04***	3.52**	Trt	5	33.64***	14.06***	5.40**	38.72***	50.53***	59.32***
Soil	2	2.74^ns^	2.56^ns^	2.77^ns^	6.80*	12.06**	6.9*	Rep	3						
Trt* Soil	10	0.97^ns^	3.39**	3.94***	3.07**	4.59***	1.32^ns^	Error	15						
Rep	9							Total	23						
Error	30							R^2^		0.89	0.83	0.77	0.93	0.92	0.94
Total	59							CV (%)		6.25	6.75	13.52	6.44	8.50	5.65
R^2^		0.79	0.84	0.78	0.88	0.89	0.74								
CV (%)		6.58	6.78	10.22	13.15	13.1	7.14								

**Table 5:** Wheat and tef growth and yield parameters as influenced by phosphorus source fertilizers and N optimization.

Treatments	Wheat						Tef					
	Plant height (cm)	Spike length (cm)	Fertile tiller number	Grain yield (kg ha^− 1^)	Biomass yield (kg ha^− 1^)	HI (%)	Plant height (cm)	Panicle length (cm)	Fertile tiller (plant)	Grain yield (kg ha^− 1^)	Biomass yield (kg ha^− 1^)	HI (%)
Control	72.3^b^	6.07^b^	2.59^b^	1898^b^	4245^b^	45.1^a^	54.5^b^	22.4^b^	4.1^b^	1376^b^	3512^b^	39.1^a^
NPS + 100%RN	98.3^a^	8.19^a^	3.83^a^	5002^a^	11,881^a^	42.1^ab^	91.9^a^	30.2^a^	6.3^a^	2477^a^	10,296^a^	24.1^b^
DAP + 100%RN	97.5^a^	8.10^a^	3.93^a^	4668^a^	12,090^a^	39.6^b^	89.9^a^	29.4^a^	4.6^ab^	2572^a^	10,432^a^	24.7^b^
TSP + 100%RN	95.4^a^	8.06^a^	3.88^a^	4640^a^	11,889^a^	39.9^ab^	91.9^a^	30.8^a^	4.9^ab^	2409^a^	9541^a^	25.2^b^
TSP + 100%RN (0 + 2/3 + 1/3)	99.7^a^	8.05^a^	4.15^a^	5350^a^	13,444^a^	40.3^ab^	97.3^a^	33.6^a^	5.4^ab^	2591^a^	10,554^a^	24.5^b^
TSP + 75%RN (0 + 1/2 + 1/2)	98.3^a^	7.97^a^	3.60^a^	4719^a^	11,829^a^	40.5^ab^	86.8^a^	30.5^a^	6.0^a^	2382^a^	9519^a^	25.2^b^
Signficance	***	***	***	***	***	**	***	***	**	***	***	***
CV (%)	6.58	6.78	10.22	13.3	13.13	7.14	5.83	0.81	13.24	6.44	8.5	5.65

***Significant at 0.1% and ** significant at 5%.

### Grain and biomass yield of wheat and tef

**Fig. 4:**
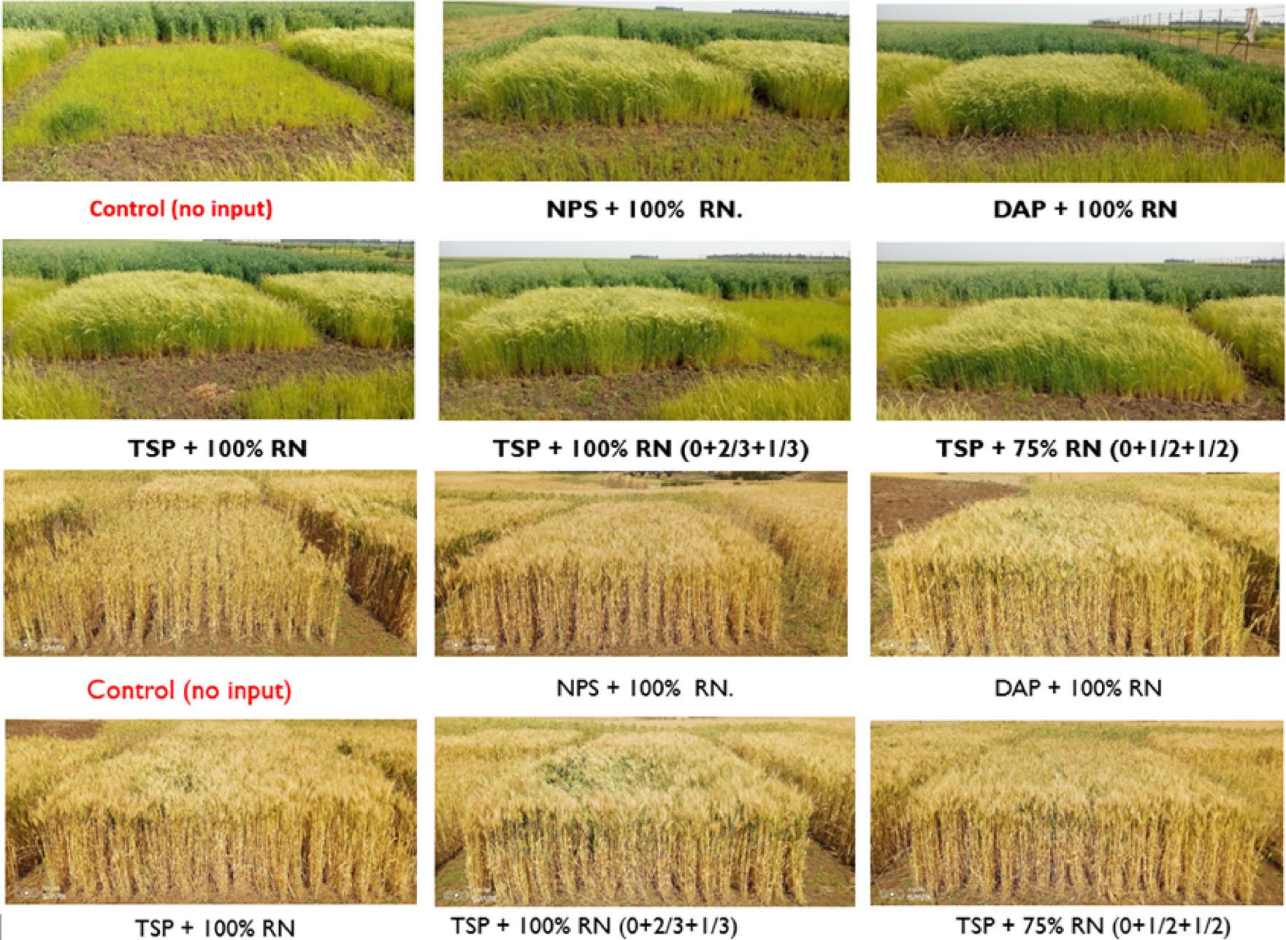
Treatment performance of tef and wheat crops in the Moretina Jiru and Basona Worana districts, respectively.

The response of wheat and tef crops to different phosphorus fertilizer sources (NPS, DAP, and TSP) and nitrogen optimization strategies was evaluated during the 2024 cropping season across farms, with farmer fields used as replications (Fig. 4). The statistical analysis of variance revealed that both wheat and tef grain and biomass yields were very significantly affected by the treatments (*p* < 0.001), as shown in Tables 4 and 5. For both crops, all fertilized treatments significantly increased grain and biomass yields compared with the control (no input) across the study sites, indicating a clear positive response to fertilizer application. However, no significant yield differences were observed among the phosphorus sources (NPS, DAP, and TSP) or the nitrogen rates (100% vs. 75% N rates) applied at different times, although the response to N timing appeared more pronounced than to the P source. This suggests that, under the slightly acidic soils with medium available P at the experimental sites, all P sources supplied sufficient plant-available phosphorus, resulting in comparable crop responses. Similarly, the comparable yields from the 75% and 100% N rates indicate that optimized split application enhanced nitrogen use efficiency, enabling lower N inputs without reducing productivity.

The mean grain yield of wheat ranged from 1898 kg ha^-1^ without fertilizer to 4640 to 5360 kg ha^-1^ across the fertilized plots, with relatively consistent performance across locations (Table 5). Similarly, the biomass yield substantially increased, ranging from 4245 kg ha^-1^ without fertilizer to 11,829 to 13,444 kg ha^-1^ in the fertilized plots. Similarly, for tef, the mean grain yield ranged from 1376 to 2591 kg ha^-1^ across all treatments, including control, showing consistent responses across locations. To evaluate how the increase in biomass translated into grain production, harvest index (HI) was also assessed. The highest HI values were recorded under the control plots (45.1% for wheat and 39.1% for tef), while fertilized treatments produced slightly lower HI values, ranging from 39.6 to 42.1% in wheat and 24.1 - 25.2% in tef. The lower HI under fertilized treatments indicates that added nutrients stimulated greater biomass production than grain formation. However, grain yield still increased markedly, showing that the reduced HI was due to elevated total biomass rather than lower productivity. Consistent HI across treatments indicates that the phosphorus source or nitrogen management did not affect assimilate partitioning.

Despite their statistical similarity, numerical variations were observed between the fertilized treatments. The highest grain yield of wheat (5360 kg ha^-1^) and tef (2591 kg ha^-1^) was recorded from the recommended rate of P_2_O_5_ from the TSP + 100% recommended N (0 at sowing + 2/3 at tillering + 1/3 at jointing). In this experiment, treatments 4 to 6, which all used TSP as the phosphorus source but with varying nitrogen rates and application timings, resulted in comparable yields. The lack of significant yield differences between full-rate and 75% nitrogen applications in both wheat and tef suggests that split nitrogen application was effective, enabling a reduction in nitrogen inputs without compromising yield, and that the application of nitrogen at planting was less important. Reducing nitrogen input also has a positive impact by lowering nitrate leaching, N₂O emissions, and the risk of water pollution, promoting more sustainable farming. This finding is particularly important for resource-constrained smallholder farmers, as it can lower input costs and reduce the environmental risks associated with excessive nitrogen use.

Similarly^48^, confirmed that DAP, MAP, and TSP, which are fertilizer sources, do not differ from each other in terms of soybean grain and biomass yields. Similarly, in a four-year experiment^49^, reported that fertilizers with different phosphorus sources (MAP, DAP, Poly P, and Ca-Mg P) had nonsignificant effects on the yield of wheat and maize. Additionally, previous studies by^50,51^ reported no statistically significant differences in yield among different phosphorus fertilizer sources. In contrast^52,53^, reported a significant yield difference between different phosphorus source fertilizers. Moreover, the unique result of this experiment is that increasing the split application of N is very important for improving nutrient use efficiency with comparable yields. Consistent with this finding^47^, reported the highest wheat grain yield from a more split application of nitrogen. Similarly^54^, reported that split application of nitrogen significantly increased the grain yield of tef compared with full N application at once.

### The effects of soil type on grain and biomass yield responses of wheat and tef to applied treatments

**Fig. 5:**
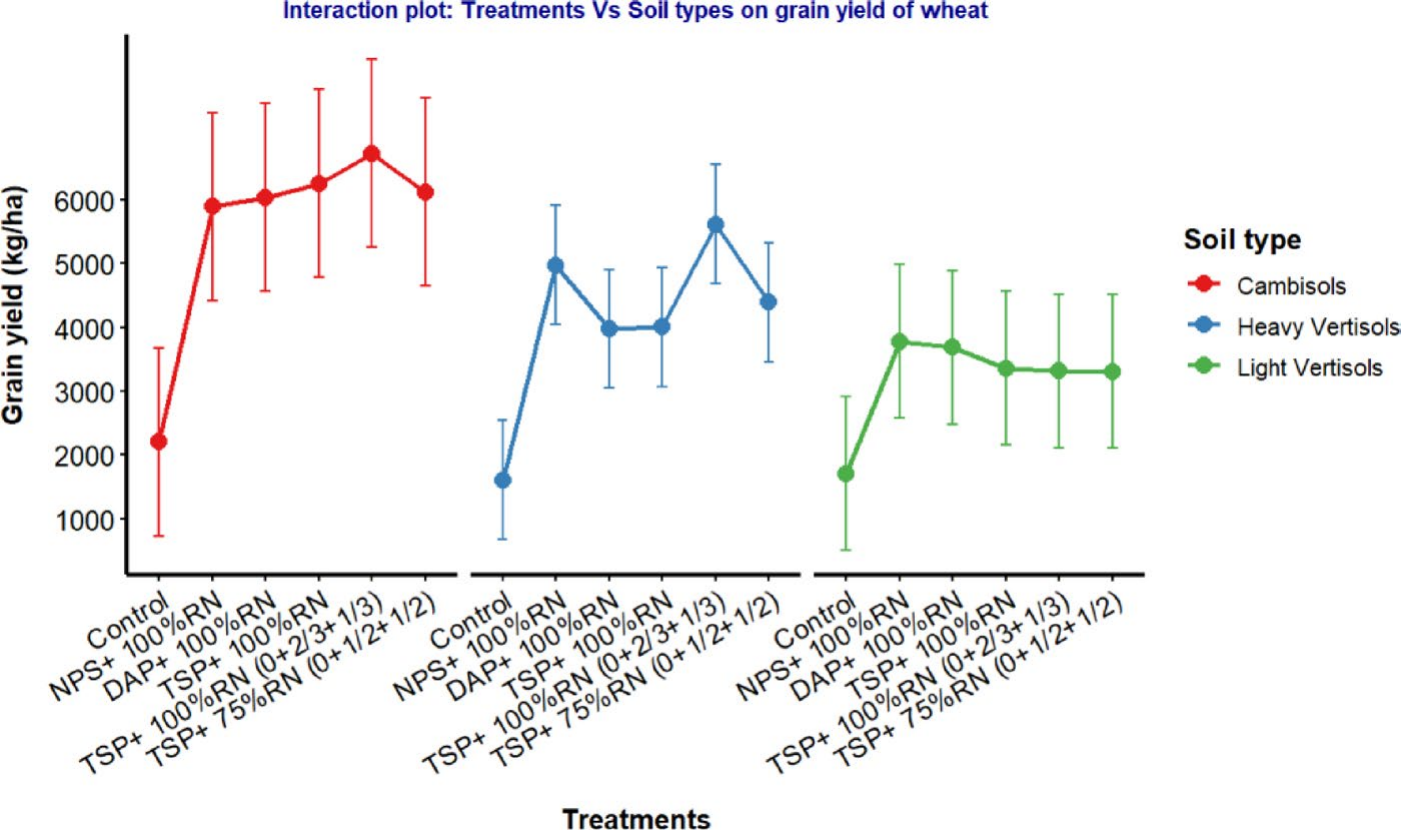
Interaction effects of treatments and soil types on the grain yield of wheat.

Among the contrasting soil types, Cambisols exhibited the highest wheat grain yield of 5631 kg ha^− 1^ and biomass yield of 14,910 kg ha^− 1^. This was followed by heavy Vertisols, while light Vertisols consistently produced the lowest yields (Fig. 5; Table 6). The superior performance of Cambisols indicates a stronger response to fertilizer applications compared to Vertisols^55^, highlighting their better soil structure, moderate to good drainage, and relatively higher fertility^56^, which together promote more efficient nutrient uptake and better root development^57^. Vertisols, which often suffer from poor drainage and compaction^58^, recorded low crop yields. Similarly^55^, reported that Cambisols showed high grain and straw yields compared with Vertisols. However, there was no significant difference between phosphorus source fertilizers on the grain and biomass yields across the soils, which implies that these fertilizer sources can be used alternatively in all the soils in our study areas.

### Interaction effects of phosphorus sources with soil type on wheat

The combined statistical analysis of variance presented in Table 4 revealed that the interaction effect of treatment and soil type highly significantly affected grain yield at *p* < 0.01 and biomass yield at *p* < 0.001. There was a clear difference in the grain and biomass yields of wheat due to the interaction of treatments and soil types. The highest mean grain yield of 6885 kg ha^− 1^ and biomass yield of 18,478 kg ha^− 1^ were observed in the Cambisols with the application of the recommended rate of P_2_O_5_ from the TSP + 100% recommended N (0 at sowing + 2/3 at tillering + 1/3 at jointing) (Table 6). These findings indicate that wheat benefited from both the Cambisol soil type and the split application of nitrogen. Similarly^55,59^, reported that the grain and straw yields of wheat were greater in Cambisols than in Vertisols because of better soil nutrient availability. In another study^57^, reported a greater response of grain and biomass yields to applied P fertilizer in Cambisols than in Vertisols. This suggests that Cambisols, which typically have better soil structure and nutrient-holding capacity, are more responsive to fertilization than Vertisols^56^. However, the lowest grain yield of 1667 kg ha^− 1^ and biomass yield of 3492 kg ha^− 1^ were recorded from the control treatment on the heavy Vertisols. The lower yield response of vertisols could be due to poor structure, aeration, and drainage problems in comparison with those of Cambisols^58^. However, the key focus of this study was to evaluate whether different phosphorus sources perform differently across soils. Our results revealed no significant differences in grain and biomass yields among the various phosphorus sources within each soil type, suggesting that these phosphorus fertilizers performed similarly regardless of the soil characteristics.

**Table 6:** Wheat grain and biomass yield response to treatments across soil types.

Treatments	Grain yield				Biomass yield			
	Cambisols	Heavy Vertisols	Light Vertisols	Mean	Cambisols	Heavy Vertisols	Light Vertisols	Mean
Control	2258	1667^c^	1768^b^	1898^b^	4972	3492^c^	4271^b^	4245^b^
NPS + 100%RN	6000	5103^ab^	3901^a^	5002^a^	14,489	11,277^ab^	9878^a^	11,881^a^
DAP + 100%RN	6124	4082^b^	3797^a^	4668^a^	17,394	8964^b^	9912^a^	12,090^a^
TSP + 100%RN	6344	4117^b^	3458^a^	4640^a^	17,444	8892^b^	9331^a^	11,889^a^
TSP + 100%RN (0 + 2/3 + 1/3)	6835	5795^a^	3421^a^	5350^a^	18,478	12,700^a^	9156^a^	13,444^a^
TSP + 75%RN (0 + 1/2 + 1/2)	6227	4509^ab^	3421^a^	4719^a^	16,683	9512^ab^	9292^a^	11,829^a^
Mean	5631^a^	4212^ab^	3294^b^		14,910^a^	9140^b^	8640^b^	
CV (%)		17.44	13.17			18.65	13.79	

### Nitrogen use efficiencies of wheat

**Fig. 6:**
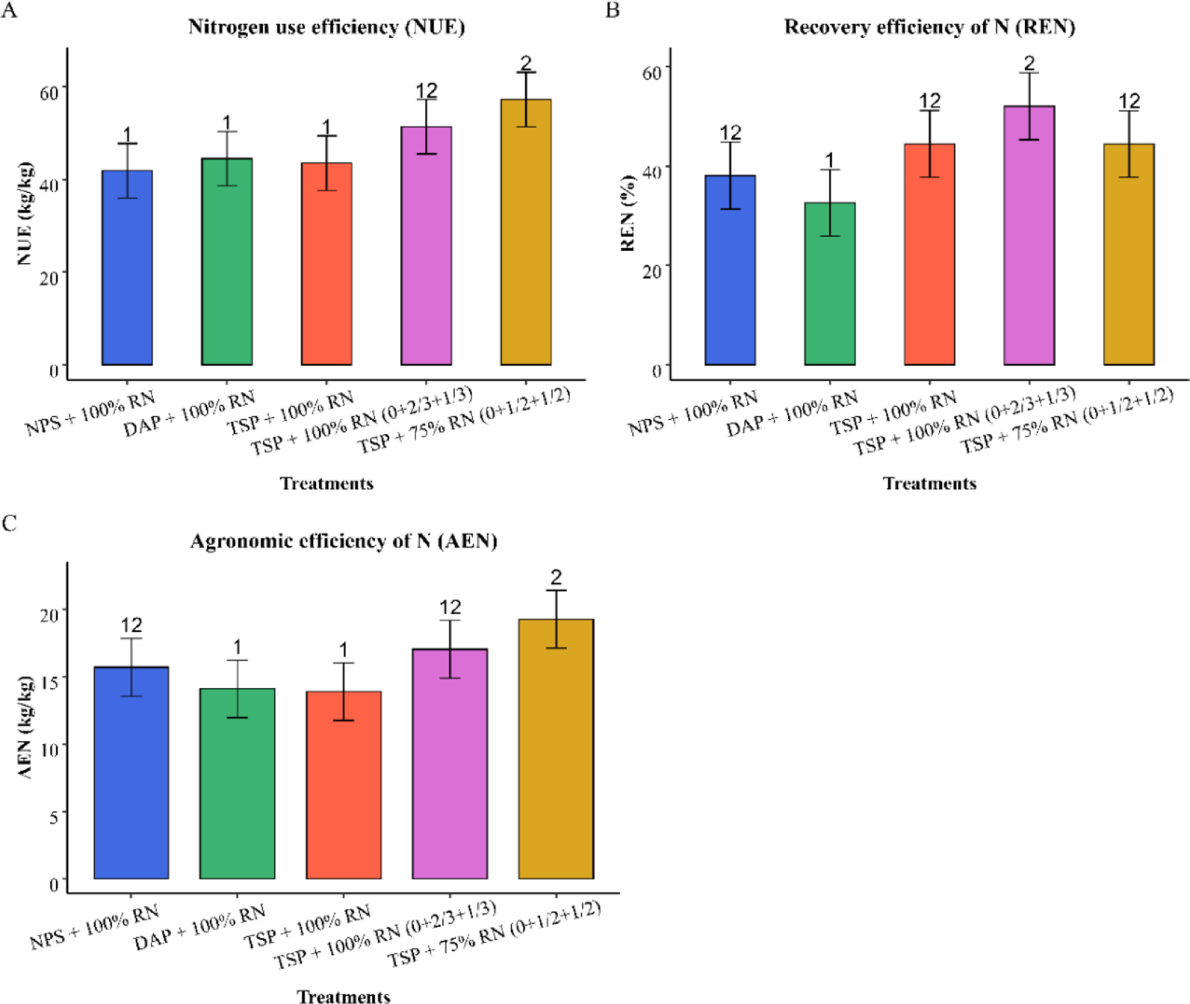
Wheat nitrogen use efficiency as influenced by treatment (*n* = 60).

Nutrient use efficiency (NUE) was evaluated for wheat but not for tef because of logistical limitations. Agronomic efficiency (AEN) is the grain yield increase per unit nutrient applied, apparent recovery efficiency (REN) is the nutrient uptake increase per unit applied, and NUE is the grain yield increase per unit nutrient taken up. The analysis of variance results showed treatments were highly significant for AEN and NUE (*p* < 0.001) and significant for REN (*p* < 0.01). Figure 6 further shows that the nitrogen optimization treatments (T5 and T6) significantly affected the nitrogen use efficiency parameters. The application of TSP + 75% RN (0 at sowing + 1/2 at tillering + 1/2 at jointing) had the highest AEN (19.30 kg kg^-1^), REN (44%), and NUE (57.2 kg kg^-1^), significantly outperforming the other treatments. This treatment improved AEN by 38.8% and NUE by 19.5% compared with the two split TSP + 100RN (1/2 at sowing + 1/2 at tillering). TSP + 100% RN (0 at sowing + 2/3 at tillering and 1/3 at jointing) also exhibited the highest REN (52%). This indicated that the nitrogen optimization treatments resulted in a greater yield increase per unit of nitrogen applied. Similarly^60^, reported increases in AEN ranging from 6.5 to 11.4% and in REN ranging from 8.4 to 14.7% as a result of split nitrogen application. Similarly^61^, reported that applying 120 kg N ha^-1^in three splits increased NUE, with a 131% increase in REN compared with two splits^62^. also confirmed that three split applications of nitrogen increased AEN by 9.33% and 10.78% over two consecutive years. However, different phosphorus sources did not affect the nitrogen use efficiency parameters (T2 to T4).

### Farmer adoption barriers and constraints of recommended fertilizer practices

Although farmers in the study generally perceived positive responses of wheat and tef to the applied phosphorus and nitrogen management practices, several factors may limit the adoption of these recommendations. Economic constraints are key, as the cost of fertilizers and labor for split nitrogen applications can be prohibitive for resource-limited smallholder farmers. Limited knowledge and awareness can also hinder adoption, as farmers may be familiar with traditional practices and may not fully understand the benefits of split nitrogen application or that different phosphorus sources can provide similar yields. Furthermore, insufficient extension support, lack of demonstration plots, and limited training opportunities may reduce farmers’ understanding of the benefits, affecting their willingness to change established practices.

In areas such as Moretina Jiru and Siyadebrena Wayu districts, where farmers traditionally apply N and P fertilizers at high combined rates (up to 1200 kg ha^-1^), adopting and disseminating the recommended practices may be particularly challenging. Additionally, the varying market prices of different phosphorus sources (NPS, DAP, TSP) and the costs associated with nitrogen fertilizers pose economic barriers. Addressing these issues through targeted training, demonstration plots, improved access to inputs, and extension support can enhance adoption, ensuring that farmers can realize both the agronomic and environmental benefits of the recommended phosphorus and nitrogen management strategies.

## Conclusion

The findings of this study underscore the importance of nitrogen optimization and balanced phosphorus application for improving the yields of wheat and tef, as well as enhancing nutrient use efficiency in wheat. No significant yield differences were observed among the phosphorus sources (NPS, DAP, and TSP) or nitrogen management strategies, indicating that all P fertilizers were similarly effective across all study soils and could be used alternatively. Notably, the comparable performance of the 75% nitrogen rate with split application highlights a cost-effective way to improve nitrogen use efficiency. These findings suggest that farmers can select phosphorus sources based on cost and availability without compromising yield, and can reduce nitrogen inputs by 25% through optimized split application, offering both economic and environmental benefits. Further research is needed on the following: (1) long-term residual impacts of DAP, NPS, and TSP on the soil and crop yield response; (2) increasing observations by catering to wider areas with additional rainy seasons; and (3) critically revising the timing of nitrogen fertilizer application across diverse environments to support wider adoption and optimize yield and nutrient use efficiency.

## Data Availability

The data sets used during the current study are available from the corresponding author on reasonable request.

## Declarations

### Permission to perform the experiment

This experiment was performed in accordance with the Debre Birhan Agricultural Research Center and Amhara Region Agricultural Research Institute review protocol. Based on this annual review, it has permission to do the experiment on the farms. The study involved on-farm fertilizer trials with volunteer farmers in North Shewa, Ethiopia. All participants gave informed consent for participation and for publication of photographs, and no personal or identifiable information was collected or disclosed.
